# Is painless colonoscopy associated with trainee learning? A single-center study of insertion speed and adenoma detection curves in 1,769 procedures

**DOI:** 10.3389/fmed.2026.1965959

**Published:** 2026-10-05

**Authors:** Yiming Ding, Hongjuan Wang, Xiangchun Lin

**Affiliations:** 1 Department of Gastroenterology, Peking University International Hospital, Beijing, China; 2 Digestive Endoscopy Center, Peking University International Hospital, Beijing, China

**Keywords:** adenoma detection rate, cecal intubation time, colonoscopy, learning curve, sedation, training quality assessment

## Abstract

**Objective:**

To compare the learning curves of cecal intubation time and adenoma detection rate (ADR) between painless (sedated) and conventional (non-sedated) colonoscopy performed by trainees.

**Methods:**

This single-center retrospective cohort comprised the first 1,769 colonoscopies performed by eight trainees without prior colonoscopy experience, grouped by sedation status into a conventional group (*n* = 852) and a painless group (*n* = 917). Mixed-effects models characterized the learning trajectories of cecal intubation time and ADR, and an extended model allowing a random case-order slope tested individual heterogeneity. The caseload to independent cecal intubation competency was estimated with a rolling 20-procedure success-rate threshold and Bernoulli cumulative sum (CUSUM) analysis.

**Results:**

Cecal intubation time was shorter in the painless group (median 6 vs. 10 min, *P* < 0.001). The case-order × procedure-type interaction fell from −0.113/SD (*P* < 0.001) in the random-intercept model to −0.027/SD (*P* = 0.589) in the random-slope model and was not significant under an exact trainee-level permutation test (exact *P* = 0.23); the overall shortening of intubation time with painless colonoscopy was robust to all specifications. Sedation status, calendar era, and scope model were highly correlated at the trainee level (*r* = 0.92), precluding separation of their independent effects. The overall ADR was 13.0% and declined early with experience (odds ratio 0.80/SD, *P* = 0.002). Fewer procedures were required to reach independent cecal intubation competency in the painless group (rolling threshold 37 vs. 114; CUSUM 107 vs. 134); however, this difference could not be separated from calendar era and equipment upgrades.

**Conclusion:**

Intubation time was shorter overall under sedation, but the faster learning of the painless group vanished once individual heterogeneity was modeled, and ADR showed no robust improvement. Sedation, era, and equipment were confounded; no causal inference regarding the effect of sedation on learning can be drawn from these data, and prospective confirmation is required. Intubation speed alone should not serve as a benchmark of overall colonoscopy competence.

## Introduction

Colonoscopy is central to colorectal cancer screening and to the diagnosis and treatment of early lesions, and its quality determines whether disease is detected in time (1, 2). Cecal intubation rate, withdrawal time, and the adenoma detection rate (ADR) are the core, widely accepted indicators of procedural quality (3, 4). Reaching an independent and stable level of competence, however, typically requires a considerable caseload, with published estimates ranging from the low hundreds to several hundred procedures (5–7). How to shorten this learning curve without compromising quality remains a central problem in endoscopy training.

Sedated (“painless”) and non-sedated (“conventional”) colonoscopy are both used in endoscopy training, each with its proponents. Sedation relieves patient discomfort and relaxes the bowel, giving trainees comparatively stable operating conditions, whereas the real-time feedback of an awake patient may shape learning differently (8, 9). How the two modes relate to a novice's trajectory remains unsettled. Case difficulty also bears on learning efficiency: female sex, older age, prior abdominal surgery, and poor bowel preparation are known to prolong insertion and increase difficulty, with anatomical and post-surgical factors further influencing intubation (10–12).

Earlier studies of the colonoscopy learning curve have largely examined a single dimension—insertion time and intubation success, or adenoma detection—seldom placing speed and detection within the same descriptive framework (13). A gap remains: during training, do gains in speed accompany parallel gains in detection? The question matters because speed and thoroughness need not improve together; if the two evolve at different rates, reaching the cecum faster is an incomplete benchmark of training success.

We therefore conducted a retrospective cohort analysis among trainees who had completed gastroscopy training and then begun learning colonoscopy. We described the learning curves of insertion speed and adenoma detection, characterized factors associated with insertion difficulty, and estimated the caseload to independent cecal intubation competency. We therefore explored whether trainees who begin under sedation (painless) conditions show a different pace of skill acquisition; because sedation status, calendar era, equipment, and trainee identity were near-collinear in our cohort, we treated the comparison as descriptive—characterizing early training trajectories and generating hypotheses—rather than estimating the causal effect of sedation.

## Materials and methods

### Study design and population

Records were drawn from the colonoscopy database of the Digestive Endoscopy Center at Peking University International Hospital. Eight trainees were included who independently performed or led first-time colonoscopies between 1 January 2016 and 31 October 2025. All eight were gastroenterology physicians who had passed gastroscopy certification and had no prior colonoscopy experience. Exclusion criteria were inadequate bowel preparation precluding completion, bowel obstruction preventing cecal intubation, missing insertion time, and missing or unverifiable key variables. Of 1,861 exported records, 51 were excluded for inadequate preparation, 18 for obstruction, and 23 for missing insertion time, leaving 1,769 cases for analysis (Figure 1). The protocol was approved by the institutional biomedical ethics committee (approval 2025-KY−0106-01); the retrospective design and full de-identification of the data waived the requirement for individual informed consent.

**Figure 1:**
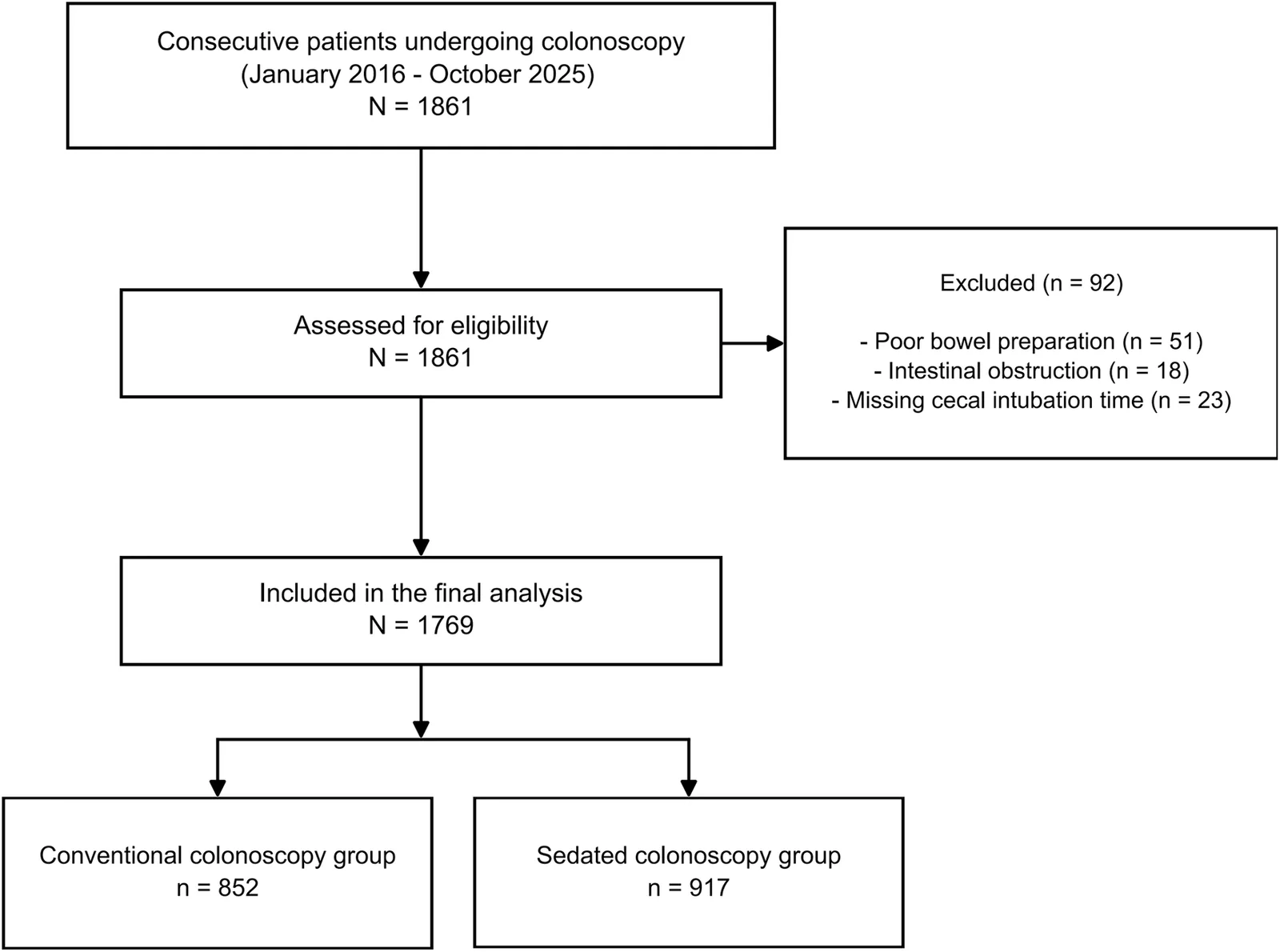
Patient enrollment and grouping flowchart. Of 1,861 exported records, 92 were excluded (inadequate bowel preparation 51, bowel obstruction 18, missing insertion time 23), leaving 1,769 cases for analysis: 852 in the conventional group (no sedation) and 917 in the painless group (under anesthesia). All tables and models are grouped by this actual sedation status; trainees 1–4 practiced predominantly conventional cases and trainees 5–8 predominantly painless cases.

Colonoscopes included the Fujifilm 590, 600, 601, and 700 series (Fujifilm, Tokyo, Japan) and the Olympus 290 series (Olympus, Tokyo, Japan). Models with variable-stiffness adjustment and optimized insertion-tube compliance (Fujifilm 601/700, Olympus 290) were classified as advanced scope models, and the Fujifilm 590/600 as basic scope models.

Procedures were grouped by the actual sedation status of each case: conventional (no sedation, *n* = 852) or painless (under anesthesia, *n* = 917). This grouping was carried through all tables, figures, and models. A descriptive secondary grouping was also defined according to each trainee's predominant mode of practice: trainees 1–4 (849 of 921 cases without sedation) were predominantly conventional, and trainees 5–8 (845 of 848 cases under anesthesia) were predominantly painless; 75 cases (4.2%) crossed modes. The two cohorts entered training 3–5 years apart, coinciding with the department's transition from basic to advanced scope models. At the case level, sedation status and calendar era (before vs. after 2021)—two binary indicators—were highly correlated, because the two generations of trainees corresponded respectively to conventional/painless practice and to basic/advanced equipment. Era, scope model, and trainee cohort were therefore strongly confounded with sedation status, and the independent effect of sedation itself could not be isolated.

### Procedure and supervision workflow

Each colonoscopy was led by a trainee from scope insertion through withdrawal. A supervising attending physician was present throughout and oversaw the procedure in real time; when insertion proved difficult (e.g., looping, fixed angulation, or failure to progress), the supervisor took over the insertion phase and advanced the scope to the cecum. The withdrawal phase (during which the mucosa is inspected and adenomas are identified) was uniformly performed by the trainee, including in cases where the supervisor had completed insertion. This workflow applied equally to the conventional and painless groups. Insertion time was recorded from scope insertion to cecal intubation regardless of whether the supervisor took over the insertion; for the 282 procedures with takeover, the recorded time therefore includes the attending's portion and is not a pure measure of trainee performance.

### Variables and outcomes

The primary outcome was cecal intubation time (the time from scope insertion to cecal intubation, referred to here as insertion time; minutes), log-transformed for analysis. Secondary outcomes were the adenoma detection rate (ADR), the polyp detection rate (PDR), the cecal intubation rate, and the rate of supervisor assistance. Cecal intubation was defined at two levels: independent intubation by the trainee (reaching the cecum without supervisor assistance) and final intubation (reaching the cecum either independently or after supervisor assistance). Trainee-independent cecal intubation was the endpoint for the competency analyses. The adenoma detection rate (ADR) was defined as the proportion of procedures with at least one histologically confirmed conventional adenoma (tubular, tubulovillous, or villous). Procedures in which cancer was detected without a synchronous adenoma were not counted as adenoma-positive. All 763 procedures in which a polyp was detected had a pathology report on record.

Covariates comprised age, sex, prior abdominal surgery, adequacy of bowel preparation, indication (screening/asymptomatic vs. symptomatic), scope model, and procedure type. Prior abdominal surgery was entered in the models as a binary variable with unknown history (*n* = 259) classified as negative; its descriptive prevalence in Table 1 is reported among patients with known history (*N* = 1,510). In approximately 2021, the center replaced the Aronchick scale (1 = excellent to 5 = poor; lower is better) (14) with the Boston Bowel Preparation Scale (BBPS, 0–9; higher is better) (15) for rating bowel preparation. Scale-specific thresholds (Aronchick 1–3, BBPS ≥ 6) were used to generate a unified binary adequate/inadequate variable; because the two scales differed, this bowel-cleanliness variable was not compared across groups. Experience was represented by each endoscopist's cumulative case order (Z-standardized; 1 SD ≈ 68 cases).

**Table 1:** Baseline characteristics of patients, grouped by actual sedation status.

Variable	Overall (*n* = 1,769)	Conventional (*n* = 852)	Painless (*n* = 917)	*P*
Age, mean ± SD, years	49.1 ± 13.3	51.1 ± 14.4	47.2 ± 11.8	<0.001
BMI, mean ± SD, kg/m^2^	24.6 ± 3.5	24.9 ± 3.6	24.4 ± 3.5	0.045
Female, *n* (%)	786 (44.4)	345 (40.5)	441 (48.1)	0.002
Prior abdominal surgery, *n* (%)	260 (17.2)	118 (18.5)	142 (16.3)	0.291
Indication—screening/asymptomatic, *n* (%)	375 (21.2)	166 (19.5)	209 (22.8)	0.1
Adequate bowel preparation, *n* (%) (scale by era)	1,594 (90.1)	802 (94.1)	792 (86.4)	—
Scope model—basic, *n* (%)	1,337 (75.6)	837 (98.2)	500 (54.5)	<0.001
Cecal intubation time, min, median (IQR)	8 (5–12)	10 (7–15)	6 (4–9)	<0.001
Trainee-independent cecal intubation, *n* (%)	1,487 (84.1)	674 (79.1)	813 (88.7)	<0.001
Polyp detection, *n* (%)	763 (43.1)	390 (45.8)	373 (40.7)	0.034
Adenoma detection, *n* (%)	230 (13.0)	148 (17.4)	82 (8.9)	<0.001

Continuous variables are mean ± SD or median (IQR); categorical variables are *n* (%). *P* values are from the t-test, *χ*^2^ test, or Mann–Whitney U test. Adequate bowel preparation was rated with different scales by era (Aronchick 1–3 adequate in the early period; BBPS ≥ 6 adequate in the later period) and is therefore not compared across groups. BMI was missing in 487 cases (466 conventional, 21 painless). Percentages for prior abdominal surgery exclude 259 unknown cases; denominator *N* = 1,510 (638 conventional, 872 painless). Cecal intubation is reported at two levels: trainee-independent (no supervisor takeover) and final (reaching the cecum after supervisor assistance).

TR, time ratio; ADR, adenoma detection rate; IQR, interquartile range.

### Statistical analysis

Continuous variables are summarized as mea*n* ± standard deviation (SD) or median (interquartile range [IQR]), and categorical variables as frequency (%); groups were compared with the t-test, *χ*^2^ test, or Mann–Whitney U test. All multivariable models used the endoscopist as a random intercept, and two-sided *P* < 0.05 was considered statistically significant.

#### Learning-curve models

The primary analysis used two mixed-effects models, with insertion time (linear mixed model) and adenoma detection rate (logistic regression, logit link) as the outcomes. Fixed effects comprised case order (linear and quadratic terms), procedure type (conventional/painless) and its interaction, and the relevant covariates: the insertion-time models adjusted for age, sex, bowel-preparation adequacy, and scope model, with prior abdominal surgery and indication omitted on the basis of their non-significance for insertion time, whereas the adenoma-detection models adjusted for the full covariate set, including indication and prior abdominal surgery. To avoid dependence on a single “common curve” assumption, the learning trajectories were tested for sensitivity to model structure: the adenoma-detection model was additionally fitted with a random-slope model allowing the case-order slope to vary across trainees and with restricted cubic splines, whereas the structural sensitivity of the insertion-time model was addressed through the random-slope robustness check described below.

#### Robustness check for the insertion-time learning curve

Building on the primary model (M0, endoscopist as random intercept), an extended model (M1) added a random slope for case order to the random-effects structure (∼ 1 + ord_z | endoscopist). Model fit was compared between M0 and M1 with a likelihood-ratio test (2 degrees of freedom difference). Because the sample contained only eight trainees, M1 was fitted with five optimizers (lbfgs, powell, bfgs, cg, nm) to ensure reliable parameter estimates, and the estimates were checked for mutual consistency with variance components within plausible bounds. As an additional sensitivity analysis, the insertion-time learning-curve models were refitted after excluding the 282 supervisor-assisted procedures, restricting the outcome to trainee-independent insertions (*N* = 1,487).

#### Few-cluster robustness analyses

Because the study included only eight trainees (four per predominant practice group) and the main between-group comparison is therefore essentially at the trainee level, four additional analyses that do not rely on model-based standard errors for cluster-level effects were performed. First, an exact randomization test: the trainee group labels were permuted exhaustively over all 70 possible 4-vs.-4 assignments and the model was refitted for each assignment; the group indicator in these analyses was each trainee's predominant practice mode (agreement with actual case-level sedation, 1,694/1,769 procedures; 95.8%). Second, a trainee-level summary analysis compared per-trainee median insertion times and per-trainee learning slopes (unadjusted and covariate-adjusted ordinary least-squares slopes, and the M1 best linear unbiased prediction slopes) between groups with exact Mann–Whitney U and exact permutation tests. Third, the primary models were refitted leaving out each trainee in turn. Fourth, a parametric bootstrap with 500 replicates simulated from the fitted random-intercept model compared model-based and empirical standard errors for the two group-level coefficients. Intraclass correlation coefficients (ICCs; on the latent response scale for the logistic model) were reported for each learning-curve model to quantify the magnitude of clustering.

#### Caseload to independent cecal intubation competency

Competency was assessed by two predefined methods. The primary method used a rolling threshold: within each trainee's cumulative sequence, a sliding window of the most recent 20 procedures was evaluated, and competency was declared at the first case at which the window's trainee-independent intubation success rate reached ≥90% (i.e., ≥18 of 20). The 90% level corresponds to the all-examinations minimum in the ASGE/ACG (American Society for Gastrointestinal Endoscopy/American College of Gastroenterology) quality indicators (16), which set a cecal-intubation target of ≥90% of all examinations and ≥95% for screening in healthy adults; it is applied here to trainee-independent intubation, which is a stricter endpoint than the procedural benchmark itself; the 20-procedure window was chosen to smooth case-to-case fluctuation while retaining localization of the crossing point. The sensitivity analysis used a Bernoulli CUSUM configured with the acceptable success rate at 90% (the same benchmark) and the unacceptable rate at 80%, with a decision interval h = 2.5, and one-sided testing for improvement toward independent cecal intubation competency. The unacceptable rate of 80% represents a 10-percentage-point decrement from the benchmark—materially substandard performance that the test is designed to detect—and the decision interval h = 2.5 lies within the range conventionally used for Bernoulli CUSUM monitoring of trainee success, toward its sensitivity-favoring end (earlier detection was preferred over occasional premature signals). Competency analyses were performed within each trainee's predominant mode of practice (conventional or painless).

#### Secondary analyses

Factors associated with prolonged insertion time were explored with a multivariable mixed-effects model, and calendar era was added in a sensitivity check. The speed–detection relationship was decomposed into within- and between-endoscopist components, estimating the association between variation in procedure time within the same endoscopist and adenoma detection, and the association between mean procedure time across endoscopists and detection.

#### Missing data

Missingness was quantified for each variable (Supplementary Table S8). Body mass index (BMI) was missing in 27.5% of procedures (*n* = 487; 466 conventional, 21 painless), and prior abdominal surgery was unavailable in 259 procedures; all other model covariates were complete. Because missingness in BMI was substantial and unevenly distributed between groups, BMI was excluded from the primary multivariable models; a complete-case sensitivity analysis additionally adjusted for BMI and did not change the main predictions. No values were imputed; prior abdominal surgery was entered with unknown history classified as negative in the adenoma-detection model (*N* = 1,769; Table 2), whereas the insertion-time full-covariate model excluded the 259 unknown-history procedures (*N* = 1,510; Table 3). All analyses were performed in Python 3.12 with the statsmodels library.

**Table 2:** Learning-curve models for insertion time and adenoma detection (multivariable mixed-effects models).

Variable	Insertion time M0 *β* (95% CI)	M0 TR (95% CI)	M0 *P*	Insertion time M1 β (95% CI)	M1 TR (95% CI)	M1 *P*	ADR M0 β (95% CI)	ADR M0 OR (95% CI)	ADR M0 *P*
Case order (Z)	+0.002 (−0.038, +0.041)	1.00 (0.96–1.04)	0.932	−0.022 (−0.111, +0.067)	0.978 (0.90–1.07)	0.622	−0.23 (−0.37, −0.08)	0.80 (0.69–0.92)	0.002
Case order^2^ (Z^2^)	−0.004 (−0.031, +0.024)	1.00 (0.97–1.02)	0.787	+0.014 (−0.016, +0.044)	1.014 (0.98–1.04)	0.356	+0.06 (−0.05, +0.16)	1.06 (0.95–1.18)	0.29
Procedure type (painless vs. conventional)	−0.188 (−0.334, −0.041)	0.83 (0.72–0.96)	0.012	−0.328 (−0.459, −0.197)	0.72 (0.63–0.82)	<0.001	−0.25 (−0.48, −0.02)	0.78 (0.62–0.98)	0.04^a^
Order × procedure type	−0.113 (−0.169, −0.057)	0.89 (0.84–0.94)	<0.001	−0.027 (−0.124, +0.071)	0.97 (0.88–1.07)	0.589	+0.17 (−0.06, +0.39)	1.18 (0.94–1.48)	0.15
Age (per 10 years)	0.04 (0.02, 0.06)	1.04 (1.02–1.06)	<0.001	—	—	—	0.64 (0.54, 0.75)	1.90 (1.72–2.11)	<0.001
Female	0.168 (0.116, 0.221)	1.18 (1.12–1.25)	<0.001	—	—	—	−0.30 (−0.53, −0.07)	0.74 (0.59–0.93)	0.011
Inadequate bowel preparation	0.122 (0.033, 0.211)	1.13 (1.03–1.23)	0.007	—	—	—	−0.79 (−1.38, −0.21)	0.45 (0.25–0.81)	0.008
Advanced scope model	−0.093 (−0.181, −0.005)	0.91 (0.83–0.99)	0.038	−0.089 (−0.175, −0.003)	0.91 (0.84–1.00)	0.042	−0.33 (−0.70, +0.04)	0.72 (0.50–1.04)	0.078

The insertion-time models (M0 and M1) use the endoscopist as a random effect and adjust for age, sex, adequacy of bowel preparation, and scope model; prior abdominal surgery and indication were non-significant for insertion time (Table 3) and were omitted. The adenoma-detection model adjusts for the full covariate set—age, sex, prior abdominal surgery, bowel-preparation-adequacy, indication (symptomatic vs. screening/asymptomatic), and scope model; excluding indication changed the case-order and procedure-type estimates by no more than 1.6% (Supplementary Table S4). Analytic sample size: *N* = 1,769 for all models in this table. Intraclass correlation coefficients: 0.10 (M0) and 0.06 at mean experience in M1 (range 0.03–0.26) for log insertion time; 0.09 on the latent scale for adenoma detection. M0 is the random-intercept model (linear mixed model for insertion time; mixed-effects logistic regression with logit link for ADR, cross-checked with a generalized estimating equation [GEE] for cluster-level standard errors). M1 is the extended model with a random slope for case order which significantly improved fit (LR = 20.84, df = 2, *P* < 0.001; slope variance *σ*^2^ = 0.0091); M1 reports only the learning-curve and procedure-related terms, as the estimates for age, sex, and bowel preparation changed negligibly between M0 and M1.

a The effect of procedure type (painless) on ADR was unstable at the cluster level (mixed model *P* = 0.04; GEE *P* = 0.17).

β, regression coefficient; TR, time ratio; OR, odds ratio; CI, confidence interval.

**Table 3:** Factors associated with cecal intubation time (full-covariate linear mixed-effects model).

Variable	β (95% CI)	*P*	Time ratio (95% CI)
Age (per 10 years)	0.041 (0.019, 0.064)	<0.001	1.04 (1.02, 1.07)
Sex (female vs. male)	0.175 (0.117, 0.234)	<0.001	1.19 (1.12, 1.26)
Prior abdominal surgery (yes vs. no)	−0.005 (−0.083, 0.072)	0.892	0.99 (0.92, 1.07)
Bowel preparation (inadequate vs. adequate)	0.114 (0.017, 0.210)	0.021	1.12 (1.02, 1.23)
Indication (symptomatic vs. screening)	0.028 (−0.040, 0.095)	0.420	1.03 (0.96, 1.10)
Scope model (advanced vs. basic)	−0.137 (−0.227, −0.047)	0.003	0.87 (0.80, 0.95)
Procedure type (painless vs. conventional)	−0.347 (−0.495, −0.199)	<0.001	0.71 (0.61, 0.82)

The endoscopist was included as a random effect with all of the above variables. Model fit: between-endoscopist variance 0.016, ICC 0.050, marginal R^2^ 0.140, conditional R^2^ 0.182. Difficult insertion (>10 min) occurred in 429/1,510 cases (28.4%), and the discriminative AUC for the above factors was 0.698 (95% CI 0.671–0.724). After calendar era was added, the painless effect attenuated toward the null (TR 0.71 → 0.82; *P* < 0.001 → 0.007).

TR, time ratio; CI, confidence interval; ICC, intraclass correlation coefficient; AUC, area under the curve. Analytic sample size: *N* = 1,510 (259 procedures with unknown prior abdominal surgery were excluded).

## Results

### Cohort and baseline

Of 1,861 exported records, 92 were excluded, leaving 1,769 cases: 852 in the conventional group and 917 in the painless group (Figure 1). Each trainee contributed 149–268 cases. Trainees 1–4 began during 2016–2018 and practiced predominantly conventional cases, whereas trainees 5–8 began after 2021 and practiced predominantly painless cases; the two cohorts were separated by 3–5 years and spanned the transition from basic to advanced scope models, so that sedation status, calendar era, equipment, and trainee identity were strongly confounded. At the case level, the correlation between sedation status and era (before vs. after 2021) was 0.92.

Baseline characteristics appear in Table 1. Mean age was 49.1 ± 13.3 years, and the painless group was younger than the conventional group (47.2 vs. 51.1 years, *P* < 0.001). Women accounted for 44.4% of the cohort overall and were more frequent in the painless group (48.1% vs. 40.5%, *P* = 0.002). The indication distribution was similar between groups (screening/asymptomatic 19.5% [166/852] in the conventional group vs. 22.8% [209/917] in the painless group; chi-square = 2.70, *P* = 0.10). Adequate bowel preparation was recorded in 90.1% overall; because the rating scale changed between eras, this variable was not compared across groups. Basic scope models predominated in the conventional group (98.2% basic vs. 54.5% basic in the painless group). Median cecal intubation time was 8 (5–12) min—10 min in the conventional group and 6 min in the painless group (*P* < 0.001). The trainee-independent cecal intubation rate was 84.1% (79.1% conventional vs. 88.7% painless); supervisor assistance was required in 282 cases, all of which reached the cecum. The overall ADR was 13.0% (230/1,769), higher in the conventional group than in the painless group (17.4% [148/852] vs. 8.9% [82/917], *P* < 0.001); the PDR was 43.1%. Individual-trainee ADRs (Supplementary Table S1) ranged from 4.5% (trainee 5) to 22.1% (trainee 3), with conventional-group trainees generally higher, consistent with their older case mix. The ADR varied slightly with the denominator used (Supplementary Table S2).

### Insertion time: learning curve, associated factors, and random-slope sensitivity

#### Learning curve

In the random-intercept-only model (M0), insertion time in the painless group declined with experience whereas the conventional group was essentially unchanged (Table 2, Figure 2A1). The conventional-group slope was flat (*β* =  + 0.002/SD, *P* = 0.932); the case-order × painless interaction was negative (*β* = −0.113/SD, 95% confidence interval [CI] −0.169 to −0.057, *P* < 0.001), giving a combined slope for the painless group of approximately −0.111/SD. Predicted curves started both groups at about 8.3 min: the conventional group remained at 8.3 min, while the painless group fell to about 5.3 min, a reduction of roughly 36%.

**Figure 2:**
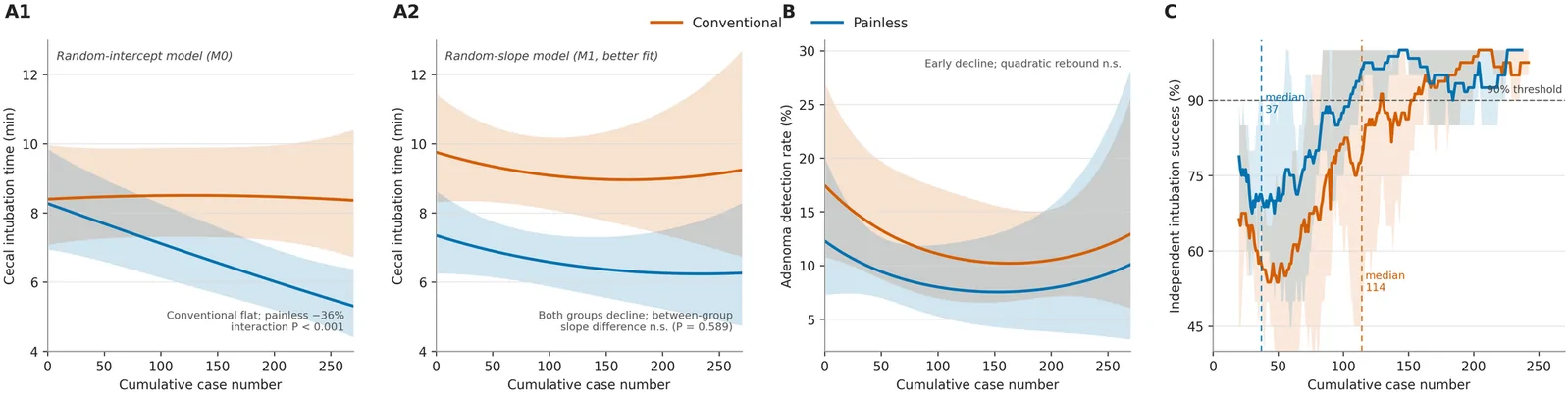
Insertion performance, adenoma detection, and intubation competency by experience. **(A1)** Cecal intubation time (minutes): marginal predicted curves with 95% CIs for the conventional and painless groups from the random-intercept model (M0); the modeled group difference is close to zero at the start of training (about 8.3 min in both groups) and widens with experience, the conventional group remaining near 8.3 min and the painless group falling to about 5.3 min (a reduction of roughly 36%; case-order × procedure-type interaction −0.113/SD, *P* < 0.001). **(A2)** The same predictions from the random-slope model (M1), which provided a significantly better fit (LR = 20.84, *P* < 0.001): both groups decline, the between-group slope difference is markedly attenuated and no longer statistically significant (interaction −0.027/SD, *P* = 0.589), and the groups differ mainly in overall level, which is present from the first case. Panels **(A1)** and **(A2)** use identical axes to allow direct comparison; their differing group baselines reflect the two models' parameterizations of the same data rather than different observed starting values—in M0 the large fitted interaction (−0.113/SD) offsets the group main effect at low case numbers, whereas in M1 the interaction is small and non-significant, so the group difference appears as an approximately constant level shift. The contrast between the panels illustrates why the between-group slope difference seen in the random-intercept model is not treated as a reliable finding. **(B)** Adenoma detection rate (ADR): marginal predicted curves with 95% CIs for the two groups; an early decline is visible, but the quadratic rebound was non-significant and not robust to model structure. **(C)** Rolling 20-procedure success rate (%) of trainee-independent cecal intubation against cumulative case number, for the conventional and painless groups, with the 90% threshold shown as a dashed line; success rose with experience in both groups (conventional 63% → 96%, painless 77% → 95%), and the painless group crossed 90% earlier (median 37 vs. 114 cases). Predicted curves in panels **(A1)** and **(A2)** are adjusted for age, sex, adequacy of bowel preparation, scope model, and procedure type, from linear mixed-effects models with the endoscopist as a random intercept (M1 additionally allowing a random case-order slope); panel **(B)** is from a logistic regression model with standard errors clustered on the endoscopist, adjusted for the full covariate set—age, sex, prior abdominal surgery, bowel-preparation-adequacy, indication (symptomatic vs. screening/asymptomatic), scope model, and procedure type.

#### Associated factors

Within the same model, the following factors were independently associated with longer insertion time (Table 3): each 10-year increase in age (time ratio [TR] 1.04, 95% CI: 1.02–1.07), female sex (TR: 1.19, 1.12–1.26), and inadequate bowel preparation (TR: 1.12, 1.02–1.23). Advanced scope models (TR: 0.87, 0.80–0.95) and painless colonoscopy (TR: 0.71, 0.61–0.82) were associated with shorter insertion time. After calendar era was added, however, the painless effect attenuated toward the null (TR: 0.71 → 0.82, *P* < 0.001 → 0.007); advanced scope models appeared almost exclusively in later painless cases and overlapped almost completely with era: 98.2% of the conventional group used basic scope models, compared with 54.5% of the painless group. Sedation status, scope model, and era were highly correlated at the trainee level (*r* = 0.92), and the independent contribution of any one factor could not be reliably separated in these data.

#### Random-slope sensitivity analysis

Extending the random intercept to a random slope significantly improved model fit (likelihood ratio [LR] = 20.84, df = 2, *P* < 0.001; slope variance *σ*^2^ = 0.0091, SD = 0.095), indicating genuine variation in learning rate across trainees. M1 converged to the same log-likelihood under all five optimizers (lbfgs, powell, bfgs, cg, nm), with consistent parameter estimates; the “parameter near boundary” warning arose from the small number of clusters (*N* = 8), and the estimates were considered reliable (convergence diagnostics in Supplementary Table S3). Extending the model nonetheless changed the between-group slope comparison: the case-order × procedure-type interaction fell from −0.113 in M0 (TR: 0.89, 95% CI: 0.84–0.94, *P* < 0.001) to −0.027 in M1 (TR: 0.97, 0.88–1.07, *P* = 0.589)—a reduction of roughly three-quarters, no longer significant (Figure 2A1 vs. Figure 2A2). In other words, the between-group difference observed in M0, in which the painless group appeared to learn faster, disappeared once individual slopes were allowed to vary. By contrast, the overall shortening of insertion time with painless colonoscopy held in both models and was stronger in M1 (TR 0.83 → 0.72, *P* = 0.012 → <0.001); the effect of advanced scope models likewise persisted (TR: 0.91, *P* ≈ 0.04). Individual-trainee slopes varied substantially: within the conventional group were both the fastest decliner (trainee 2, −0.073) and trainees whose time rose with experience (trainees 3 and 4, +0.110 and +0.162), a span of 0.236; within the painless group the variation was smaller (−0.097 to +0.025, span 0.122; Supplementary Figure S1, Table S3).

#### Few-cluster robustness

The exact trainee-level permutation test (all 70 assignments; no failed fits) confirmed the overall group effect on insertion time (predominant-mode grouping: TR: 0.62, exact *P* = 0.029 in M0; TR: 0.61, exact *P* = 0.014 in M1, the smallest attainable value) but not the learning-speed difference (case-order × group interaction, exact *P* = 0.23), although the model-based Wald test in M0 had yielded *P* < 0.001 (indicating that the model-based Wald test was anti-conservative for this cluster-level effect). At the trainee level, per-trainee median insertion times were 10.0 vs. 6.25 min (exact Mann–Whitney *P* = 0.029, the smallest attainable for four vs. four), whereas per-trainee slopes were directionally consistent but non-significant (exact permutation *P* = 0.17–0.20). Leave-one-trainee-out refits left the overall group effect unchanged (TR: 0.78–0.84 in M0; 0.63–0.76 in M1) while the interaction varied more than twofold (−0.08 to −0.17). A 500-replicate parametric bootstrap gave standard errors consistent with the model-based values (0.065 vs. 0.075 for the group effect; 0.029 vs. 0.028 for the interaction) with negligible bias, indicating that the fragility of the interaction arises from between-trainee slope heterogeneity rather than from unstable estimation. These analyses are summarized in Supplementary Table S5. Restricting the analysis to mode-consistent procedures only (excluding the 75 crossover cases) did not change either conclusion (group effect, exact *P* = 0.029; interaction, exact *P* = 0.13). The clustering itself was modest: the ICC was 0.10 for log insertion time in M0 (between-trainee variance 0.033; residual variance 0.303), 0.06 at mean experience in M1 (0.03–0.26 across the experience span), and 0.09 for adenoma detection on the latent scale; so procedure-level estimates were little affected by clustering, unlike the trainee-level contrasts.

#### Independent-insertion sensitivity analysis

Because the recorded insertion time includes the attending's portion for the 282 supervisor-assisted procedures (takeover rate 20.9% conventional vs. 11.3% painless), the learning-curve models were refitted on the 1,487 trainee-independent insertions (674 conventional, 813 painless; all eight trainees contributed). The overall group-level shortening of insertion time remained robust (random-intercept model TR 0.80, 95% CI: 0.69–0.93, *P* = 0.003; random-slope model TR: 0.69, 0.61–0.78, *P* < 0.001; exact trainee-level permutation *P* = 0.029). The between-group slope difference again failed the robustness checks: the case-order × procedure-type interaction was −0.085/SD (*P* = 0.006) in the random-intercept model but fell to −0.014/SD (*P* = 0.76) in the random-slope model, and it was not significant under exact permutation (exact *P* = 0.34), mirroring the full-cohort pattern. The advanced-scope-model association was essentially unchanged in magnitude (TR: 0.92–0.93, vs. 0.91–0.92 in the full cohort) but was no longer significant in the smaller restricted sample (*P* = 0.09–0.11). Because exclusion of takeover procedures may itself introduce informative selection, the all-procedures analysis remains primary (Supplementary Table S6).

Taken together, the overall shortening of insertion time with painless colonoscopy was robust across models, whereas the between-group slope difference—“the painless group learns faster than the conventional group”—was sensitive to model specification, significant in the random-intercept model yet markedly attenuated once individual slopes were allowed to vary (Figure 2A1 vs. Figure 2A2) and not significant under exact trainee-level permutation, and is therefore insufficient as a reliable conclusion.

### Collinearity of sedation, scope model, and era

The three factors—sedation status, calendar era, and equipment model—were nearly collinear at the trainee level, with pairwise correlations all exceeding 0.9: the painless and conventional groups were enrolled 3–5 years apart, and advanced scope models were concentrated in the painless group. When calendar era (split at 2021) was added as a covariate, the TR for painless colonoscopy rose to 0.82 and its *P* value changed from <0.001 to 0.007; advanced scope models overlapped almost completely with era and could not be estimated independently.

The caseload to independent cecal intubation competency was reached earlier in the painless group, and the between-group gap narrowed when CUSUM was used instead; here too the gap could not be disentangled from era and equipment.

### Adenoma detection rate

Table 2 and Figure 2B present the trajectory of the adenoma detection rate. The odds ratio for case order was 0.80/SD (*P* = 0.002), indicating an early decline. The quadratic term had an odds ratio (OR) of 1.06/SD (*P* = 0.29). In the restricted cubic splines, the linear-decline component had an OR of 0.87 (*P* = 0.07) and the nonlinear component *P* = 0.21. In the random-slope model the slope variance approached zero, model fit did not improve over the random-intercept model, and the quadratic term was again non-significant (*P* ≥ 0.23). Individual trajectories differed considerably (Supplementary Figure S2) and did not follow a single common curve. The association between sedation and adenoma detection was fully absorbed once era was added (OR rising from 0.78 to 1.01; era itself OR: 0.62, *P* < 0.001; Supplementary Table S4), a direction consistent with the sensitivity of insertion time to era.

The case-order × procedure-type interaction was not significant (*P* = 0.15), and decomposing the speed–detection association into within- and between-endoscopist components offered no compensatory signal: within the same endoscopist, cases whose procedure time exceeded that endoscopist's own mean had only a borderline-lower ADR (OR: 0.97/min), and the between-endoscopist association was null (OR: 1.04/min).

### Caseload to independent cecal intubation competency

Using a rolling 20-procedure window with a ≥90% independent-intubation success-rate threshold, the median number of procedures to independent cecal intubation competency was 114 (range 72–158) in the conventional group and 37 (range 20–87) in the painless group. With CUSUM instead, the median was 134 (85–205) in the conventional group and 107 (58–131) in the painless group. Independent cecal intubation success rose with experience in both groups (Figure 2C): in the conventional group from 63% in the early phase to 96% in the late phase, and in the painless group from 77% to 95%. The two definitions differed by 20 (conventional) and 70 (painless) median cases within the same trainees, underscoring that the absolute caseload is an operational, definition-dependent quantity rather than a fixed threshold. At the stricter ≥95% rolling criterion (≥19 of 20), the median caseload was 131 vs. 80 cases, with the direction of the difference preserved (Supplementary Table S7).

## Discussion

This cohort documented how insertion speed and adenoma detection evolved with experience across the first 1,769 colonoscopies of eight trainees. The most defensible finding is that insertion time was shorter overall under painless colonoscopy, whereas older age, female sex, and inadequate bowel preparation prolonged it. Whether the painless group also learned faster is a separate matter: the random-intercept model raised the possibility, but once individual learning rates were allowed to vary, the between-group difference vanished—it behaved more like individual variation compressed into a group contrast. Adenoma detection, in turn, did not improve in step with faster insertion. The two skills did not mature together during early training, and insertion speed alone is an incomplete measure of training success.

Multivariable analysis confirmed the principal determinants of insertion difficulty: older age, female sex, and inadequate bowel preparation prolonged insertion time, whereas advanced scope models and painless colonoscopy shortened it. Painless colonoscopy was nonetheless highly correlated with calendar era and equipment at the trainee level. After era was added, its time ratio rose from 0.71 to 0.82, and its speed advantage could not be separated from “later era, better equipment.” The literature does not support anesthesia itself directly lowering insertion difficulty: propofol sedation does not raise the cecal intubation rate (17), and its benefit lies mainly in patient comfort; excessively deep sedation is instead linked to more missed adenomas (18). The shorter insertion time in the painless group is therefore better read as a composite reflection of era, scope model, and the stability of operating conditions than as an independent effect of anesthesia. The magnitude of the overall group effect also varied with model specification (TR: 0.61–0.82).

This collinearity also bounds the answer to a second question: whether sedation and advanced scope models can shorten a novice's learning curve. The directional evidence is suggestive: in M0 the painless group's insertion time fell with experience while the conventional group held flat, and the rolling-threshold caseload to independent cecal intubation competency was likewise smaller for the painless group. That evidence, however, carries an unavoidable weakness (Figure 2A1 vs. Figure 2A2). The case-order × procedure-type interaction lost significance once the random slope was added (*P* falling from <0.001 to 0.59, with the magnitude reduced by about three-quarters), even as model fit improved significantly (LR = 20.84, *P* < 0.001). The eight trainees spanned a slope range of nearly 0.24: within the conventional group were both fast learners and trainees who slowed with experience, while the painless group varied somewhat less, over a range of about 0.12. Learning rates were heterogeneous to begin with, and the random-intercept model forced that heterogeneity into a common within-group slope, so part of the individual variation was counted as a group effect. Two implications for training follow. First, “painless colonoscopy makes one learn faster” does not hold in these data; the one result consistent across M0 and M1 is the overall shortening of insertion time with painless colonoscopy. Second, learning rate shows substantial individual variation (19), and a single uniform threshold for the caseload to independent cecal intubation competency cannot fit everyone; rather than fixate on a one-size-fits-all numerical target, programs would do better to identify slow learners early and provide targeted reinforcement. Partitioning the overall speed gain among sedation, scope model, and era is beyond what these data allow: the three are almost collinear (pairwise r ≈ 0.92 at the trainee level), and only a prospective design matching sedated with conventional procedures within the same era and the same scope model can answer the question.

Caseload to independent cecal intubation competency was likewise asymmetric: under the rolling 20-procedure threshold the painless group reached independent cecal intubation competency at a median of 37 cases vs. 114 in the conventional group, whereas the stricter CUSUM pushed the milestones later overall and narrowed the gap from 77 to 27 cases. Directional agreement aside, the two methods differ in magnitude by construction, so the advantage can only stand as a hypothesis awaiting confirmation. Both figures sit below the estimates of roughly one hundred to several hundred procedures in the literature, and around 275–300 procedures when competency is defined with comprehensive tools such as the Assessment of Competency in Endoscopy (ACE) (20). Much of this gap reflects how the endpoint is defined: this study measures the single skill of independent cecal intubation, unlike the overall competence captured by comprehensive assessment tools; our trainees had all passed gastroscopy certification and brought transferable endoscopic skills to colonoscopy, and both painless conditions and advanced scope models favor intubation. What the smaller caseload here denotes is early attainment of independent intubation, not overall competence.

Adenoma detection exhibited an early linear decline (OR: 0.80 per SD of experience, *P* = 0.002), but this trend was sensitive to model specification: the quadratic term for case order was non-significant, and neither spline analysis nor the random-slope model supported a common U-shaped trajectory, with marked individual variation. We therefore report only the observed linear decline and do not infer a uniform nadir or a later rebound. One hypothesized (but untested) account is that early in training, when insertion is slow, trainees might withdraw more cautiously and inspect more meticulously, favoring detection; as experience accrues and insertion time falls, withdrawal thoroughness might not be sustained, and detection might decline before recovering as the trainee's recognition of lesion patterns matures.

This hypothesis could not be tested in the present dataset, because withdrawal time was not recorded in the large majority of procedures. That account nonetheless fits a broader pattern: withdrawal time directly affects the adenoma detection rate (21, 22); computer-aided detection raises detection only in low-baseline endoscopists (23, 24), not across the board; and deep sedation increases the miss rate. Whether the improvement comes from a better scope or from artificial intelligence, the technology on its own does not translate into higher detection. Within a single endoscopist, a one-minute difference in insertion time left the ADR essentially unchanged (OR 0.97/min), further evidence that insertion speed itself does not drive adenoma detection (25–27); given that the influence of withdrawal time on detection has been demonstrated repeatedly, the fluctuation in detection during early training may reflect differences in the meticulousness of withdrawal inspection. We therefore present this only as a possible explanation for the observed pattern, not as its likely mechanism; confirmation would require prospective measurement of withdrawal time and the withdrawing operator.

The strengths of this study include the consecutive inclusion of each trainee's complete initial cases, a uniform training workflow, and a histologically defined adenoma detection rate. Several limitations should also be noted. First, only eight trainees were included at a single center in a retrospective design, with sedation, era, equipment, and trainee identity collinear; between-group contrasts are exploratory and do not support causal inference. With only four trainees per group, a systematic difference between the two cohorts in baseline endoscopic skill—technical facility or familiarity with colonic anatomy, for instance—cannot be ruled out as residual confounding. Second, with only eight trainees, cluster-level inference is intrinsically limited: model-based standard errors for between-group effects can be anti-conservative, and exact trainee-level tests have minimal power. The exact permutation analyses (Supplementary Table S5) are therefore presented alongside the model-based results, and the between-group slope difference in the insertion-time learning curve did not survive the random-slope specification.

Third, withdrawal time was not recorded. Because in our workflow withdrawal was uniformly performed by the trainee, the proposed mechanism—that the trainee's withdrawal thoroughness varies with accumulating experience and thereby drives the early ADR decline—remains conjectural without direct withdrawal-time measurement. Accordingly, the early decline in ADR is reported as an observation whose mechanism remains unverified. Fourth, when the supervising physician took over to overcome an obstacle, the recorded time to cecal intubation included the attending's portion of the procedure, and the duration of takeover varies with the difficulty of the individual case. A sensitivity analysis restricted to independently completed insertions is reported in Supplementary Table S6; because takeover occurs preferentially in difficult procedures and was more frequent in the conventional group (20.9% vs. 11.3%), this restriction may itself introduce selection, and the primary analysis therefore retained all procedures. Fifth, the adenoma-detection trajectory rests on a few individuals; the early decline was not robust to the random-slope and spline structures and individual curves were heterogeneous, so we do not claim a common U-shaped curve or a definite nadir. Sixth, the bowel-preparation score changed from the Aronchick scale to the BBPS across eras, BMI was missing in 27.5% of cases with uneven distribution, and records of sedation depth and the agents used in the painless group were incomplete, so residual confounding by these variables and unseparated variation in sedation depth cannot be excluded. Finally, these results come from a single training center with specific equipment and two cohorts of trainees, and generalization to other training environments should be made with caution.

Because sedation practice, equipment, and trainer intervention changed in real time rather than under controlled conditions, a retrospective design cannot fully isolate the independent effect of sedation; a prospective cohort with standardized, real-time trainer intervention would be required to obtain more realistic and less biased estimates.

Overall, insertion speed can improve quickly during early training and is shorter overall under sedation, but the claim that “the painless group learns faster” does not survive a test of individual heterogeneity, and adenoma detection showed no matching robust improvement. Insertion speed is therefore unsuitable as the sole gauge of overall competency; the very phase in which insertion speed rises fastest is precisely when withdrawal inspection and adenoma surveillance deserve the most attention. A multicenter prospective cohort could test whether this divergence between speed and detection recedes as endoscopists mature; if the early decline in adenoma detection is confirmed, that phase becomes a clear target for reducing interval cancer (28, 29).

## Conclusion

In this retrospective cohort, cecal intubation time was shorter overall under painless colonoscopy, while older age, female sex, and inadequate bowel preparation were independent factors associated with prolonged insertion time. The painless group's learning curve declined more steeply in the point estimates, but this difference was unstable across statistical models and is presently insufficient to conclude that the painless group learns faster. The painless group required fewer procedures to reach independent cecal intubation competency under each method of calculation, but the absolute figure depended on how competency was defined and could not be separated from calendar era and equipment. Because sedation, calendar era, and equipment were confounded, no causal inference regarding the effect of sedation on learning can be drawn from these data. The adenoma detection rate displayed no robust improving trend with accumulated experience. Insertion speed alone cannot serve as a measure of operative competency.

## Data Availability

The raw data supporting the conclusions of this article will be made available by the authors, without undue reservation.

## Supplementary material

SUPPLEMENTARY FIGURE S1

Individual insertion-time trajectories for the eight trainees (M1 random slope). A 2 × 4 eight-panel layout, one panel per trainee. The y-axis is insertion time (minutes) and the x-axis is each trainee's cumulative case number. In each panel, points are single-case insertion times, the solid line is the 25-case rolling median (trend), and the gray dashed line is the 10-minute reference; the upper-right annotation gives the trainee's slope BLUP from M1 (change in log-time per SD of case order) and the corresponding per-case percentage change in time (↓ shorter with experience, ↑ longer with experience). Within the conventional group (trainees 1–4, red tones) the BLUP slopes span nearly 0.24-trainee 2 learned fastest, whereas trainees 3 and 4 grew slower with experience; within the painless group (trainees 5–8, blue tones) the BLUP slopes span about 0.12 and were mostly negative. The figure directly illustrates the main-text point that heterogeneity in learning rate across individuals is substantial and the group-level slope difference is not robust to the random-slope specification (values in Table S3). Each panel pools all of a trainee's cases (both sedation types); group labels follow each trainee's predominant mode (Methods).

SUPPLEMENTARY FIGURE S2

Individual adenoma detection rate (ADR) trajectories for the eight trainees. The y-axis is ADR and the x-axis is each trainee’s cumulative case number. The eight individual curves differ in shape, and the experience interval at which the nadir and rebound occur is inconsistent across trainees; no single common trajectory is apparent, supporting the statement that no common U-shaped ADR curve and no robust rebound were observed (sensitivity analysis in Table S4).
