# Supplementary material for: Is painless colonoscopy associated with trainee learning? A single-center study of insertion speed and adenoma detection curves in 1,769 procedures
Source: Front Med (Lausanne). 2026 Oct 5;13:1965959. doi: 10.3389/fmed.2026.1965959 (PMC13677516; doi:10.3389/fmed.2026.1965959)
Supplement: Supplementary file 1 [file Supplementaryfile1.docx]

**Supplementary tables**

**Table S1.** Individual characteristics and performance of the eight trainees.

| **Trainee** | **Group** | **Era (case distribution)** | **Cases** | **Trainee-independent cecal intubation (%)** | **Supervisor assistance (%)** | **Insertion time median (IQR), min** | **PDR (%)** | **ADR (%)** |
| --- | --- | --- | --- | --- | --- | --- | --- | --- |
| 1 | Conventional | 2016–2018 | 240 | 88.8 | 11.2 | 11 (8–15) | 49.2 | 18.8 |
| 2 | Conventional | 2016–2018 | 252 | 80.6 | 19.4 | 8 (5–14) | 40.9 | 19.8 |
| 3 | Conventional | 2016–2018 | 222 | 70.7 | 29.3 | 10 (7–16) | 54.5 | 22.1 |
| 4 | Conventional | 2016–2018 | 207 | 81.6 | 18.4 | 10 (7–15) | 35.3 | 6.8 |
| 5 | Painless | 2022–2025 | 268 | 89.2 | 10.8 | 6 (4–8) | 37.7 | 4.5 |
| 6 | Painless | 2021–2024 | 149 | 85.9 | 14.1 | 7 (5–9) | 55.7 | 14.8 |
| 7 | Painless | 2022–2024 | 194 | 88.1 | 11.9 | 6 (5–9) | 29.4 | 6.7 |
| 8 | Painless | 2024–2025 | 237 | 87.3 | 12.7 | 5 (4–7) | 45.1 | 10.5 |

Trainees 1–4 were predominantly conventional and trainees 5–8 predominantly painless. This table supports the statement in the main text that individual trajectories are heterogeneous and follow no single common curve.

**Table S2.** Adenoma detection rate stratified by denominator.

| **Denominator stratum** | **n** | **Adenomas** | **ADR (%)** |
| --- | --- | --- | --- |
| All procedures | 1769 | 230 | 13.0 |
| Trainee-independent intubations | 1487 | 179 | 12.0 |
| Supervisor-assisted (reached cecum after takeover) | 282 | 51 | 18.1 |

**Table S3.** Insertion-time learning rate (M1 random-slope BLUP) and convergence diagnostics for the eight trainees.

| **Trainee** | **Group** | **Intercept BLUP** | **Slope BLUP** | **Interpretation** |
| --- | --- | --- | --- | --- |
| 1 | Conventional | +0.195 | −0.003 | Slow baseline, steady learner |
| 2 | Conventional | −0.118 | −0.073 | Fast baseline, fast learner |
| 3 | Conventional | +0.177 | +0.110 | Slow baseline, slowing with experience |
| 4 | Conventional | +0.081 | +0.162 | Most pronounced slowing with experience |
| 5 | Painless | −0.111 | −0.079 | Fast baseline, fast learner |
| 6 | Painless | −0.026 | +0.025 | Slightly slow |
| 7 | Painless | −0.035 | −0.045 | Fairly fast learner |
| 8 | Painless | −0.164 | −0.097 | Fastest baseline, fastest learner |

BLUPs are from M1 (random-slope model for case order); the slope unit is the change in log insertion time per 1 SD of case order (approximately 68 cases). The conventional-group slopes span 0.236 (−0.073 to +0.162) and the painless-group slopes 0.122 (−0.097 to +0.025), so individual heterogeneity within the conventional group is about twice that of the painless group. M1 variance components: slope variance σ² = 0.0091 (SD = 0.095), intercept variance = 0.0177, intercept–slope covariance = 0.0086 (correlation ≈ +0.68), residual variance = 0.2986. Convergence diagnostics: all five optimizers (lbfgs, powell, bfgs, cg, nm) converged to the same log-likelihood (llf = −1457.262) with consistent parameter estimates; the "MLE near parameter-space boundary" warning arose from the small number of clusters (N_clusters = 8), the slope variance was non-zero, and the intercept–slope correlation did not reach the ±1 boundary, so the estimates were considered acceptable (full report in convergence_diagnostics.txt). Individual trajectories are visualized in Supplementary Figure S1.

**Table S4. Sensitivity of the ADR learning curve to calendar era, model structure, case mix (indication), and missing-data handling (unknown surgical history).**

| **Model specification** | **Key term** | **Estimate** | **P** | **Interpretation** |
| --- | --- | --- | --- | --- |
| + Era | Painless (baseline) | OR 0.78 | 0.040 | Sedation–ADR association before adjustment |
| + Era | Painless (+ era) | OR 1.01 | 0.92 | Fully absorbed by era |
| + Era | Era (after 2021) | OR 0.62 | <0.001 | Era predominates |
| Random slope | Order² (quadratic) | OR 1.06 (M0) / 1.07 (M1) | 0.29 / 0.23 | Rebound not robust to random slope |
| Random slope | Slope variance | ≈0 (degenerated) | LR P = 1.0 | Random slope does not improve fit |
| Restricted cubic splines | Linear term | OR 0.87 | 0.07 | Linear decline attenuates to borderline |
| Restricted cubic splines | Nonlinear (vs. linear) | LR 4.6, df 3 | 0.21 | No significant nonlinearity |
| Restricted cubic splines | AIC (linear/quadratic/spline) | 1235 / 1235 / 1234 | — | Three equivalent, no preferred shape |
| Individual trajectories (Figure S2) | — | Heterogeneous | — | No single common ADR curve |
| ± Indication (full-covariate model) | Indication (symptomatic vs. screening) | OR 0.83 (0.70–0.98) | 0.028 | Included a priori as a case-mix determinant; coefficient not robust across estimators (cluster-robust OR 1.02), not interpreted |
| ± Indication (full-covariate model) | Case-order OR, model without vs. with indication | 0.81 / 0.80 | 0.004 / 0.002 | Learning-curve estimate unchanged |
| ± Indication (full-covariate model) | Procedure-type OR, model without vs. with indication | 0.78 / 0.78 | 0.033 / 0.036 | Estimate unchanged |
| Stratified by indication | Mantel–Haenszel OR, painless vs. conventional | 0.45 (0.34–0.61) | Breslow–Day 0.61 | Equals the crude OR (0.46); no confounding or heterogeneity by indication |
| Direct standardization, indication × age tertile | Group ADR after standardization | 15.8% vs. 9.7% | — | Crude 17.5% vs. 8.8% (row-level reclassification); gap persists after case-mix standardization |
| Handling of unknown surgical history | Case-order OR, unknown-as-negative (N = 1,769) vs. complete-case (N = 1,510) | 0.80 / 0.86 | 0.002 / 0.063 | Early decline and procedure-type estimate (0.78/P 0.036 → 0.82/P 0.091) lose significance under restriction; unknowns concentrated in the conventional group (25.1% vs. 4.9%), making exclusion informative—consistent with the fragility of the ADR findings emphasized in the text |

**Table S5. Few-cluster robustness analyses for the trainee-level (cluster-level) effects.**

| **Analysis** | **Target effect** | **Result** |
| --- | --- | --- |
| **Exact permutation over all 70 trainee-label assignments** | **Overall group effect (painless vs. conventional), predominant-mode grouping** | **TR 0.62, exact P = 2/70 = 0.029 (M0); TR 0.61, exact P = 1/70 = 0.014 (M1, smallest attainable) — robust** |
| Exact permutation over all 70 assignments | Case-order × group interaction (learning-speed difference) | Exact P = 16/70 = 0.23 despite Wald P < 0.001 in M0 — Wald SE anti-conservative for this cluster-level effect; not robust |
| Trainee-level summary (4 vs. 4), exact tests | Per-trainee median insertion time | 10.0 vs. 6.25 min; exact Mann–Whitney P = 0.029 (smallest attainable) — robust |
| Trainee-level summary (4 vs. 4), exact tests | Per-trainee learning slopes (unadjusted / adjusted / BLUP) | Directionally consistent (painless more negative) but non-significant; exact permutation P = 0.17–0.20 — no conclusion drawn |
| Leave-one-trainee-out refits (8 ×) | Overall group effect | TR 0.78–0.84 (M0), 0.63–0.76 (M1); no single trainee drives the effect — robust |
| Leave-one-trainee-out refits (8 ×) | Case-order × group interaction | Sign constant but magnitude varied >2-fold (−0.08 to −0.17) — fragile |
| Parametric bootstrap, 500 replicates | SE calibration of the two group-level coefficients (M0) | Bootstrap SE vs. model SE: 0.065 vs. 0.075 (group); 0.029 vs. 0.028 (interaction); bias negligible — Wald machinery internally calibrated |
| Intraclass correlation coefficient (ICC) | Magnitude of clustering (insertion time M0/M1; ADR mixed logit) | 0.10 (M0); 0.06 at mean experience in M1, 0.03–0.26 across the experience span; 0.09 latent-scale (ADR) — modest clustering; level-1 inferences unaffected |
| Parametric bootstrap, 500 replicates (M1) | SE calibration of the M1 group-level coefficients | Bootstrap SE vs. model SE: 0.063 vs. 0.067 (group effect); 0.049 vs. 0.050 (interaction) — calibrated, consistent with the M0 results |

M0, random-intercept model; M1, random-slope model; TR, time ratio; BLUP, best linear unbiased prediction; SE, standard error. Exact P values are the proportion of the 70 possible 4-versus-4 trainee assignments yielding a statistic at least as extreme as the observed assignment.

**Table S6. Sensitivity analysis restricted to trainee-independent cecal intubations (excluding the 282 supervisor-assisted procedures).**

| **Term** | **Full cohort, M0 (N = 1,769)** | **Full cohort, M1 (N = 1,769)** | **Independent-only, M0 (N = 1,487)** | **Independent-only, M1 (N = 1,487)** |
| --- | --- | --- | --- | --- |
| Sample composition (conventional / painless; takeover rate) | 852 / 917; 20.9% / 11.3% | 852 / 917; 20.9% / 11.3% | 674 / 813; takeover excluded | 674 / 813; takeover excluded |
| Painless (main effect), TR (95% CI) | 0.82 (0.71–0.95), P = 0.007 | 0.72 (0.63–0.82), P < 0.001 | 0.80 (0.69–0.93), P = 0.003 | 0.69 (0.61–0.78), P < 0.001 |
| Case-order × painless (interaction), β/SD | −0.113, P < 0.001 | −0.027, P = 0.589 | −0.085, P = 0.006 | −0.014, P = 0.758 |
| Advanced scope model, TR | 0.91, P = 0.031 | 0.92, P = 0.042 | 0.93, P = 0.109 | 0.92, P = 0.088 |
| Random-slope variance (M1) | — | 0.0091 | — | 0.0084 |
| Exact trainee-level permutation, group effect | P = 0.029 (TR 0.62) | — | P = 0.029 (TR 0.58) | — |
| Exact trainee-level permutation, interaction | P = 0.23 | — | P = 0.34 | — |

Models as in Table 2 (random intercept, M0; random slope, M1), adjusted for age, sex, bowel-preparation adequacy, and scope model. Takeover procedures were slower than independent procedures (median 10 vs. 7 minutes) and more frequent in the conventional group (20.9% vs. 11.3%); because excluding them may introduce informative selection, the full-cohort analysis remains primary. TR, time ratio; β, regression coefficient; CI, confidence interval. Exact permutation tests permuted the trainee group labels over all 70 possible 4-versus-4 assignments (predominant-mode grouping, as in Table S5). The advanced-scope-model association was essentially unchanged in magnitude in the restricted analysis (TR 0.92–0.93 vs. 0.91–0.92) and lost significance consistent with the smaller sample (P = 0.09–0.11).

**Table S7. Caseload to trainee-independent cecal intubation competency under three definitions.**

| **Definition** | **Conventional, median (range)** | **Painless, median (range)** | **Difference in medians** |
| --- | --- | --- | --- |
| Rolling 20-procedure window, ≥90% (≥18/20) | 114 (72–158) | 37 (20–87) | 77 |
| Rolling 20-procedure window, ≥95% (≥19/20) | 131 (76–200) | 80 (55–108) | 51 |
| Bernoulli CUSUM (90% vs. 80%, h = 2.5) | 134 (85–205) | 107 (58–131) | 27 |

All eight trainees reached each criterion. Across increasingly conservative definitions, the median caseload rises in both groups while the between-group difference narrows monotonically (77 → 51 → 27 median cases), illustrating that the absolute caseload is an operational, definition-dependent quantity rather than a fixed threshold.

**Table S8. Missingness of analysis variables and its handling.**

| **Variable** | **Missing/unknown, n (%)** | **Handling in analyses** |
| --- | --- | --- |
| Age | 0 | Complete |
| Sex | 0 | Complete |
| Body mass index | 487 (27.5) | Excluded from the primary multivariable models because missingness was strongly differential between groups (466/852, 54.7% conventional vs. 21/917, 2.3% painless); a complete-case sensitivity analysis additionally adjusting for BMI (N = 1,282) did not change the main predictions |
| Prior abdominal surgery | 259 (14.6) unknown | Adenoma-detection model: binary with unknown history classified as negative (N = 1,769); insertion-time full-covariate model: complete-case (N = 1,510); the descriptive prevalence in Table 1 is reported among patients with known history (N = 1,510) |
| Bowel-preparation adequacy | 0 | Complete |
| Indication (screening/asymptomatic vs. symptomatic) | 0 | Complete |
| Scope model | 0 | Complete |
| Insertion time | 0 | Complete |
| Cecal intubation and supervisor takeover | 0 | Complete |
| Polyp detection and adenoma status | 0 | Complete (adenoma status not applicable when no polyp was detected; all procedures with a detected polyp had a pathology report on record) |

N = 1,769 procedures. No values were imputed; prior abdominal surgery was entered with unknown history classified as negative in the adenoma-detection model (Table 2: N = 1,769) and excluded in the insertion-time full-covariate model (Table 3: N = 1,510), and the analytic sample size is reported for each model.
